# Endovascular treatment of aortic saddle embolism through percutaneous mechanical Thrombectomy via Straub Rotarex catheter

**DOI:** 10.1186/s13019-020-01334-5

**Published:** 2020-09-29

**Authors:** Hong-Zhi Yu, Xiao-Bo Guo, Zhao Liu, Zhe Zhang, Hai Feng, Xue-Ming Chen

**Affiliations:** Department of Vascular Surgery, Beijing Friendship Hospital, Capital Medical University, No.95 Yongan Road, Xicheng District, Beijing, 100050 China

**Keywords:** Abdominal aorta saddle embolism (ASE), Endovascular treatment, Percutaneous mechanical thrombectomy (PMT), Continuous blood purification

## Abstract

**Background:**

To summarize our experience of endovascular treatment for abdominal aorta saddle embolism (ASE) through percutaneous mechanical thrombectomy (PMT).

**Methods:**

Clinical data of three ASE patients treated with an endovascular approach using percutaneous mechanical thrombectomy (PMT) were reviewed and analyzed.

**Results:**

After PMT, blood flow of limbs was restored in all of the three patients. However, two patients died from sudden cardiac arrest caused by hyperkalemia several hours after the procedure. The other one patient survived through continuous renal replacement therapy, which was initialized shortly after the surgical procedure.

**Conclusion:**

Endovascular treatment through PMT can quickly restore blood flow in the ASE patients. Blood purification through renal replacement therapy is crucial to reduce mortality after restoring blood flow of the limbs.

## Background

Peripheral arterial embolism is a common disease in the field of vascular surgery, but aortic saddle embolism (ASE) is a rare and critical disease with more severe complications and high death rate. Serious complications or even death may occur even if the ASE could be diagnosed timely and treated properly because of bifurcate embolus “striding” aorta, which may cause arterial obstruction, and serious hemodynamic and metabolic disorders in the femoral arteries in both legs [1–3]. Therefore, once ASE is diagnosed, treatment procedure should be determined immediately based on reasonable clinical judgment. Currently, thrombectomy through Fogarty catheter incision via both side femoral arteries or thrombectomy through transabdominal abdominal aorta incision are commonly used [1, 4]. Application of endovascular treatment through percutaneous mechanical thrombectomy (PMT) for ASE, however, has rarely been reported. Here, we present three cases of ASE who were treated through PMT via Straub Rotarex catheter (Sraub Medical, Switzerland).

## Methods

### Patients

**Fig. 1:**
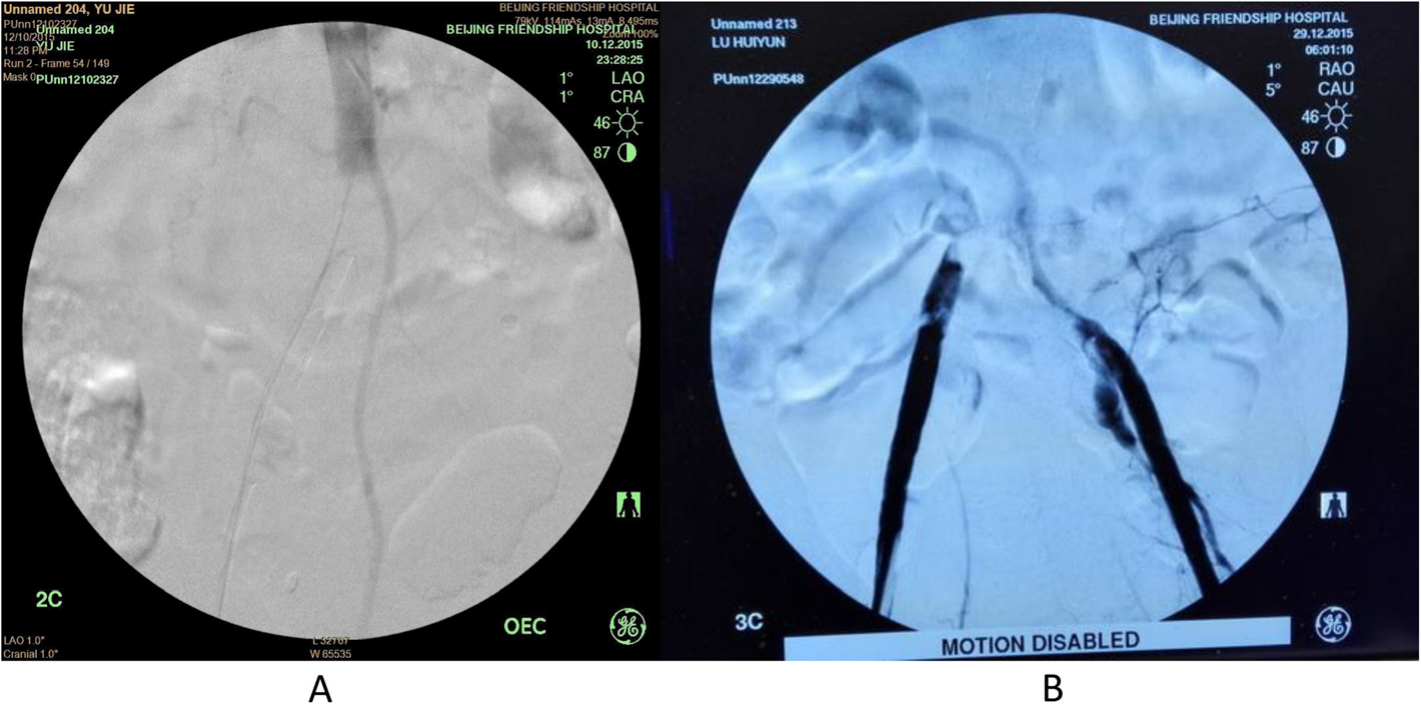
Angiogram before treatment. Panel **a**: Angiogram of Case #2, showing catheter passing through the segment of thrombus and blockage of the lower segment of the main abdominal artery. Panel **b**: Bilateral femoral artery angiogram of Case #3, showing bilateral common iliac arteries were completely filled with thrombus

Total three ASE cases (2 male and one female), who were hospitalized and treated in the Department of Vascular Surgery from October to December 2015, were retrospectively analyzed in the current study. Acute limb ischemia was classified following the Rutherford classification criteria [5]. Laboratory tests including myocardial enzyme, cardiac troponin T (TnT), and serum potassium were performed before and after the operation. Preoperative abdominal contrast-enhanced CT, arterial ultrasonic doppler of lower extremities were also performed in all three patients. As shown in Fig. 1, ASE was diagnosed in all three patients by contrast angiogram.

### Treatment

**Fig. 2:**
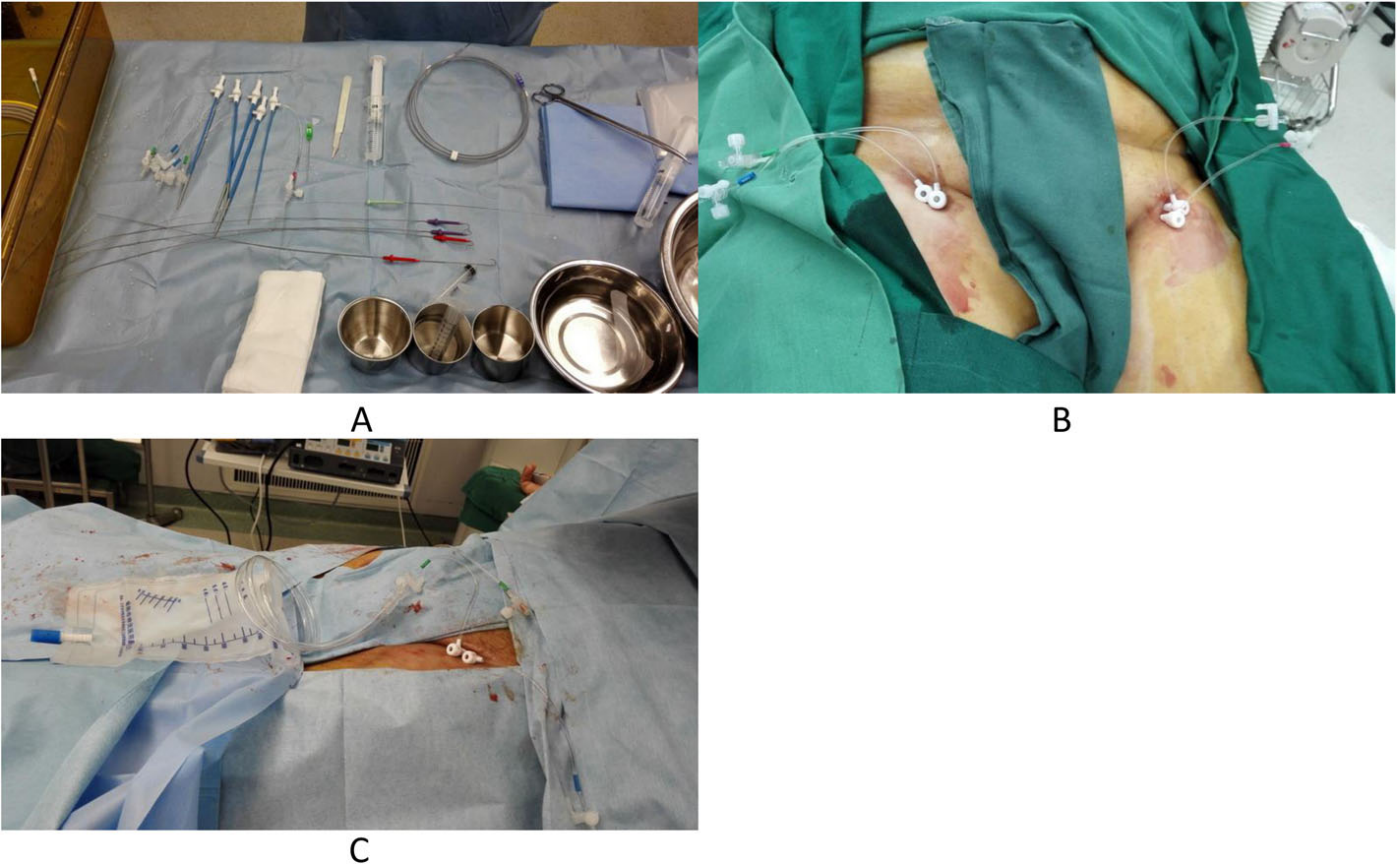
Application of sheath during PMT. Panel **a:** Sheaths. Panel **b**: Sheaths indwelled in the both side femoral arteriovenous vessels. Panel **c**: Drainage bag for collecting venous blood through the intravenous sheath

Patients were given sodium polystyrene sulfonate orally or by clyster for hyperkalemia, and were hospitalized for emergent treatment of ASE with percutaneous mechanical thrombectomy (PMT) procedure using 8F Rotarex catheter. Specifically, one patient was treated with both same side and opposite side PMT through a crossover operation by unilateral femoral artery retrograde puncture under local anesthesia as well as indwelling a catheter for thrombolysis in the abdominal aorta and unilateral iliac artery, but without indwelling venous sheath. The other two patients were treated with PMT using 8F Rotarex catheter through bilateral femoral artery retrograde puncture under general anesthesia, and balloon expanding stent implantation for stenotic segment of artery after thrombus removal. One of them was given an additional unilateral iliac artery stent, while the other patient was given an additional popliteal artery stent and bilateral femoral vein puncture sheath indwelling (Fig. 2).

To avoid absorption of metabolic toxins, bleeding through the venous sheath was conducted as opening the arteries. Total 600 mL blood (300 mL each side) was collected, washed with an autologous blood collection device, and then infused back after erythrocyte washing and purification.

After the surgical operation, the arterial puncture sites were sutured through a blood vessel suture instrument (ProGlide, Abott Company, the United States) followed by pressure dressing performance.

## Results

### General information of the patients and findings of laboratory examination

General characteristics of the three patients were listed in the Table 1. Age of the patients was from 28 to 76 years old with an average of 55 years old, and the suffering time of ASE were 10 ~ 96 h. Two of them had serious ischemia of unilateral limb, and the other one had extensive ischemia of both lower extremities.

**Table 1:** Information of the three patients

	Case #1	Case #2	Case #3
Gender	Male	Female	Male
Age (years old)	61	28	76
ASE suffering time (h)	10	96	72
Classification for acute limb ischemia	Class IIb	Class IIb	Class III
Complications			
Coronary heart disease	No	No	Yes
Hypertension	Yes	No	No
Atrial fibrillation	No	No	No
Other complications	Hyperkalemia, chronic bronchitis	No	Rheumatic heart disease, hematuria, hyperkalemia
Preoperative high TnT	Yes	No	Yes
Preoperative high myocardial enzyme	Yes	No	Yes
Preoperative ultrasonography	Blood flow at the proximal iliac artery was blocked.	Blood flow at the proximal iliac artery was blocked.	Blood flow at the proximal iliac artery was blocked.
Preoperative CT	Diagnosed as ASE	Not done	Diagnosed as ASE
Surgical anesthesia method	Local anesthesia	General anesthesia	General anesthesia
PMT treatment method	Unilateral puncture, crossover operation	Bilateral puncture	Bilateral puncture
Location of thrombus	Thrombus of lower abdominal aorta and double iliac arteries	Thrombus of lower abdominal aorta and double iliac arteries, and left popliteal artery thrombus	Thrombus of lower abdominal aorta and double iliac arteries, and right iliac artery stenosis
Combined with other endovascular treatment methods	Indwelling catheter for thrombolysis in abdominal aorta and right iliac artery	Left popliteal artery stent	Right common iliac artery stent
Intravenous indwelling of the sheath for recovery of venous blood	No	Indwelling for double femoral veins	Indwelling for double femoral veins
Volume of recovered venous blood (ml)	0	600	600
Postoperative hemofiltration	No	Yes	No
Postoperative outcome	Dead	Cured and discharged	Dead
Angiogram after PMT treatment	The thrombus disappeared, and blood supply of limbs was improved apparently.	The thrombus disappeared, and blood supply of limbs was improved apparently.	Thrombus locally attached to the wall of abdominal aorta was considered, and blood supply of limbs was improved apparently
Amputation/death	Died early after the operation	No	Died early after the operation
Osteofascial compartment syndrome	No	No	No
Occurrence of cardiopulmonary arrest	Postoperative 120 min	No	Postoperative 20 min
Time of death	4 h after surgery	/	6 h after surgery

Note: 1. *ASE* aortic saddle embolism; 2. for Case #3, families of the patient refused amputation treatment

All three patients were transferred from other hospitals. Two out of the three patients were Class IIb and one was Class III by the Rutherford classification criteria. One patient had past history of rheumatic heart disease and myocardial infarction, two patients had hypertension, and one patient was long-term ex-smoker. None of the three patients had history of atrial fibrillation.

### Outcomes of the treatment

**Fig. 3:**
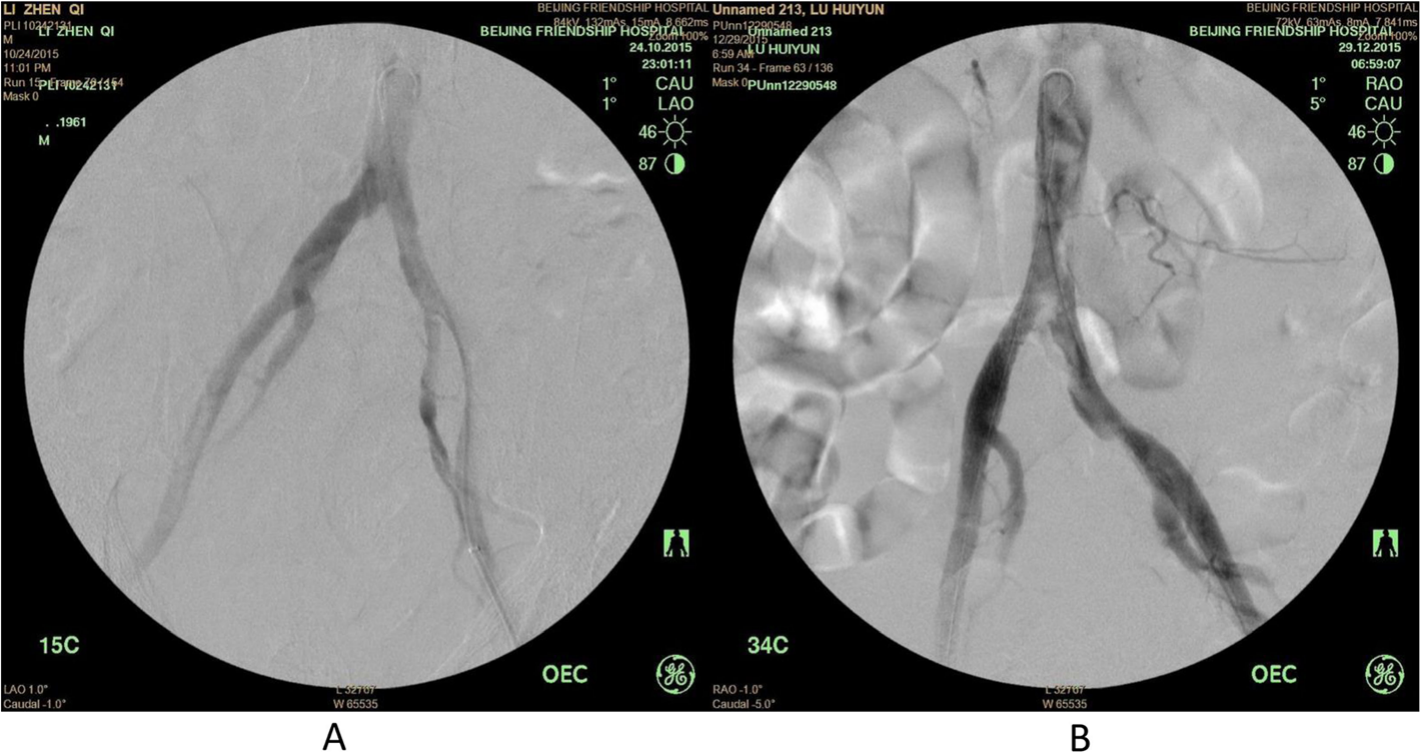
Postoperative angiogram. Panel **a**: Postoperative angiogram of Case #1, showing blood flow of abdominal aorta and iliac artery was restored. **Panel b**: Postoperative angiogram of Case #3, showing residual thrombus attached to the wall of the lower abdominal aorta, while the blood flow of iliac artery was restored

All three patients were treated with PMT within 3 h after admission to our hospital. Postoperative angiograms showed that thrombus at iliac artery disappeared in the Case #1 patient, limb blood supply improved significantly, and no distal embolism was found (Fig. 3a). However, in Case #3, a residual thrombus at the lower end of abdominal aorta was noticed, which could be the thrombus attached to the wall and was not able to be removed through multiple suction (Fig. 3b).

Thrombus was not formed at the puncture site of femoral artery. One patient (Case #2) survived after the surgical operation and bedside hemofiltration (renal replacement therapy) in the ICU. Osteofascial compartment syndrome was not found in this patient. The other two patients (Case #1 and #3) died 4 h and 6 h after surgical operation, respectively. As shown in Table 2, these two deceased patients had perioperative hyperkalemia, high myocardial enzyme and high TnT.

**Table 2:** Preoperative and postoperative laboratory test results of the three cases

Serial number	Myoglobin (ng/ml)		Creatine phosphokinase (40 ~ 200 U/L)		Creatinine (μmol/L)		Serum potassium (mmol/L)	
	Preoperative	Postoperative	Preoperative	Postoperative	Preoperative	Postoperative	Preoperative	Postoperative
Case 1	Not examined	694	60,390	63,520	185.3	231.3	5.99	7.2
Case 2	Not examined	753	4267	10,498	117.0*	265.7	3.63*	4.2*
Case 3	Not examined	> 1000	> 42,670	> 42,670	162.3	209.4	5.95	4.9*

## Discussion

### Present status of ASE treatment

Half century ago (1950–1970), death rate of ASE was 22–75%, and the main causes of death were myocardial infarction, renal failure, thrombosis or embolism recurrence, and stroke [3]. A retrospective study showed that acute aortic occlusion was caused by either embolism (65%) or thrombosis (35%). Heart disease and gender (female) were risk factors of embolism, while smoking and diabetes were risk factors of thrombosis. Mortality and comorbidity of aortic occlusion were 35 and 74%, respectively [2]. Conventional surgical treatment of ASE may cause revascularization syndrome, which results sometime in serious complications such as loss of a limb, multi-organ failure or even death due to compartment syndrome, skeletal muscle necrosis, hypovolemic shock, hyperkalemia, myoglobinuria, acute renal failure, acidosis, or arrhythmia. Therefore, early diagnosis and timely surgical operation are critical in reducing death rate and disability rate in the ASE patients [1, 6].

For ASE treatment, the following potential complications or outcomes should be considered: 1). Functional recovery of ischemic limbs; 2). Perioperative death rate and postoperative death rate; 3). Perioperative complications; and 4). Risk of embolism reoccurrence. The most common surgical procedure for ASE is thrombectomy via Fogarty catheter through femoral arteries or via abdominal aorta incision. If the patient has serious arteriosclerosis, a bypass operation such as axillary artery - femoral artery bypass or abdominal aorta - femoral artery artificial vascular bypass may be performed [1]. For patients with advanced limb ischemia (Class III of acute limb ischemia), amputation is often recommended [5]. In the current study, Case #3 had extensive necrotic plaque of lower limbs, which was classified as Class III of acute limb ischemia, and thus, amputation was recommended to the patient. However, the patient firmly refused it, and therefore, PMT treatment was performed in addition to conservative treatment including vasodilation drugs, anticoagulation drugs and sodium polystyrene sulfonate.

Whole lumen treatment in ASE has rarely been reported in the literature. In this regard, Yang et al. reported one successful case of ASE treatment with film-coated stent (Viabahn stent, W. L. Gore & Associates, the U.S.A) [7]. Specifically, the film-coated stent covered the lower abdominal aorta and bilateral iliac artery thrombus, while the distant parts of the iliac arteries from the stent were covered by an additional balloon expanding stent in order to prevent thrombus translocation [7]. This was a successful case of whole lumen treatment without reduction in thrombus volume. However, there was a risk of proximal thrombus translocation in this case, especially if it was an extensive thrombus without volume reduction. In addition, it might block blood supply to the bilateral internal iliac arteries, which could inevitably affect local blood supply and ischemia of pelvic organs and gluteus muscles.

It was also reported that combination of catheter-directed thrombolysis (CDT) and endovascular stent treatment had been successfully used to treat infrarenal abdominal aorta thrombosis or iliac artery thrombosis [8]. In this report, 11 patients were treated with CDT followed by placing a self-expandable stent at the lesion of the main iliac artery in order to prevent potential complications such as embolisms at renal artery or distal arteries. Through this procedure, the patients had significant improvement in clinical symptoms, signs, and ABI [8]. However, there was a risk of losing therapeutic opportunity if the ischemia progressively accelerated in these patients, especially in the patients with severe ASE, because CDT is a time-consuming procedure. Therefore, for the patients with severe ASE, establishing blood flow by PMT followed by CDT to remove residual thrombus might be a safer and more effective procedure.

### Innovation and improvement of surgical procedures

Currently, open operation is the most commonly used procedure for ASE although hybrid operation has also been reported [9]. With clinical application of new devices for endovascular mechanical removal thrombus, both traditional surgery and hybrid therapy face with a revolution. New devices for mechanical removal of endovascular thrombus include catheter-directed thrombus aspiration, thrombus aspiration by fragmentation or rheological thrombolysis, isolated segmental pharmacomechanical thrombolysis, and ultrasound-accelerated thrombolysis [10]. In this regard, a previous study indicated that, technically, success rate of ultrasound-accelerated thrombolysis for the treatment of aorta-femoral artery thrombotic occlusion was relatively high [11]. Application of this technology resulted in complete disappearance of the thrombus within 24 h in nearly 50% of the patients and low recurrence rate of complications within 30 days. The study further demonstrated that ultrasound-accelerated thrombolysis was safe and effective, and superior to CDT for the treatment of thromboembolism in the arteries and bypass graft vessels in the region of the abdominal aorta-femoral artery [11].

In European centers, PMT is regularly used for the treatment of acute and sub-acute thrombosis. European experience demonstrated that PMT seemed to be a feasible technology for acute and sub-acute embolism at the femoral artery bifurcates. PMT could simply and directly remove the blood clots and restore the blood flow quickly without delaying the treatment compared to the conventional operation procedure [12].

Rotarex®S catheter has been proved to be safe and effective in the treatment of peripheral artery diseases with 95% success rate [13]. Combined application of Rotarex®S catheter and Straub power system could be used for percutaneous endovascular excision of thrombus, embolism, or atherosclerotic plaques in the vessels other than cardiopulmonary, coronary, and cerebral vessels. Unlike the thrombolytic treatment, Rotarex®S catheter can be used for thrombus extraction even in the case of anticoagulation contraindication exists in the patient and results in rapid blood flow restore. Compared to thrombus extraction by Fogarty catheter through surgical operation, thrombus extraction by Rotarex catheter is a mini-invasive surgical procedure, and the percutaneous puncture technique could not only reduce the risk of distal thrombus translocation, but also the duration of surgical procedure. However, main shortcoming of Rotarex catheter is that the catheter cannot pass through a narrow lesion or smaller lumen, especially in the case of heavily calcified plaque, and there is a risk of artery perforation [14]. In the current study, residual thrombus was not completely removed from the lower end of thrombus at abdominal aorta by PMT operation in the patient #3 even after attempting by changing angles of the guide wire, suggesting Rotarex catheter could hardly reach the whole thrombus and thus, unable to completely remove the thrombus in a relatively wide abdominal aortic cavity. In patient #1 of the current study, a crossover operation technique was used. We found the angle of guide wire was relatively small at the femoral artery bifurcates, and thus, a retrograde puncture procedure at bilateral femoral arteries was used in the patient #2 and #3 in order to prevent the guide wire from breaking during the operation. In addition, general anesthesia was applied in the patient #2 and #3 because severe limb pain was noticed in these patients during the procedure.

**Fig. 4:**
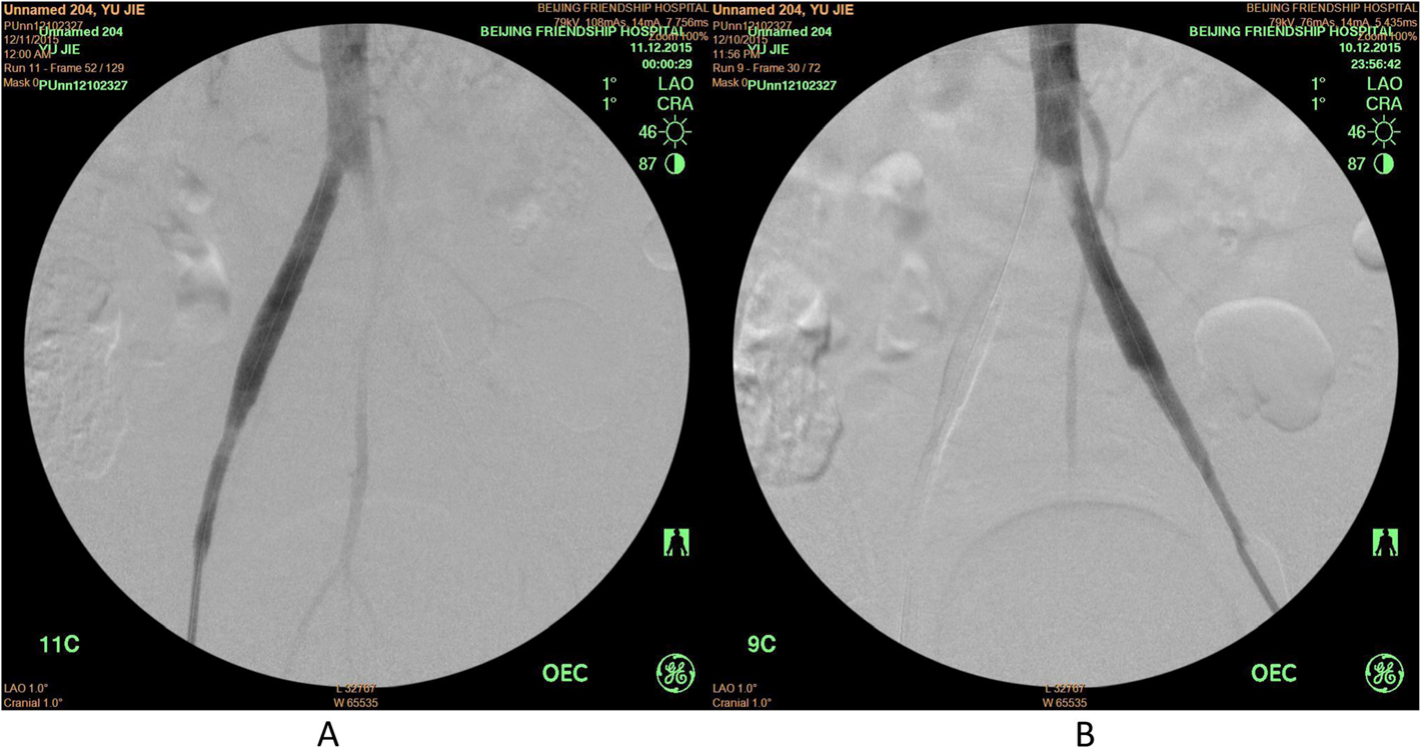
Postoperative left and right iliac artery angiogram of Case #2

Our experience of PMT therapy with Rotarex catheter indicated that the advantages of this therapy were as followings: 1). Labor saving: one physician with one assistant are sufficient to conduct the operation. 2). Short operation time: bilateral thrombus removal can be completed within 20 ~ 30 min. Generally, most of the thrombus can be removed after 2–3 times of operation, and the blood flow can be restored rapidly (as shown in Figs. 3 and 4 in the current study). In addition, suturing the puncture site with a vessel suturing device greatly shortens the time compared to the conventional surgical incision and suture time, which requires at least 1.5 h for thrombus extraction and suture of incision given two groups of personnel operate simultaneously. 3). Application of the sheath allows to perform angiography evaluation or other endovascular procedure during the operation: bilateral or crossover operation results in quick restore of the blood flow. Angiography through the sheath allows to examine the status of thrombus removal immediately after PMT procedure, which avoids the limited estimation on the thrombus removal status by blood flow rate in the artery, the complicated hybrid procedure, and repeated procedures of angiography/operation/angiography again to confirm the thrombus removal status. In addition, CDT procedure or stent implantation can also be performed through the sheath.

### Ischemia-reperfusion injury and renal replacement therapy

Recanalization following limb ischemia may result in reperfusion syndrome, which causes very high comorbidity and mortality. In this context, insufficient estimation on the risk of reperfusion syndrome before the emergent operation might contribute to the high rate of comorbidity and mortality in the elderly patients with variety kinds of complications [15]. Ischemia-reperfusion injury refers to more serious tissue damage or even irreversible lesions after the blood flow is restored. Ischemia-reperfusion injury may significantly affect the outcome of ASE treatment and it is still a challenging problem for the treatment of severe limb ischemia. In this regard, two patients in the current study died after successful PMT and blood restore even through these two patients had received hemofiltration therapy and sodium polystyrene sulfonate, suggesting that ischemia-reperfusion be alerted to save ASE patient’s life.

Following blood flow restore, large amounts of pro-inflammatory cytokines, oxygen radicals, lactic acid and potassium ions are released to the blood flow, which augments myonephropathic metabolic syndrome (MNMS). MNMS results in damage of kidney function (10–15% patients have acute kidney failure) through rhabdomyolysis and potential nephrotoxin, which cause increase of myoglobin and creatine phosphokinase (CK). Myoglobin is toxic to kidney and its half-life in the blood flow is 2 ~ 3 h, while CK is a biomarker of muscle injury and its half-life is 1.5 days. Accumulation of CK in the blood may indicate the progress of renal failure. Not surprisingly, in the current study, the two deceased patients had high serum potassium, TnT and CK before the surgery, indicating serious muscle necrosis existed before the surgery, and it became worse after restoring blood flow by surgery. This might be the direct cause of death for the two patients even though they were treated with bicarbonate and mannitol to prevent acute renal failure as well as to reverse oliguria renal failure to non-oliguria renal failure following the literature reports [3, 15, 16].

Toxic metabolites in the venous blood following ischemia-reperfusion may cause systemic complications. Therefore, bypass the venous blood from returning to systemic circulation after PMT surgery is a desirable approach to prevent ischemia-reperfusion injury. However, drainage of femoral vein blood flow may cause serious blood loss and volume consumption, which increases the risk of fatality in critically ill patients. In addition, toxic metabolite removal through draining femoral vein blood is technically difficult in clinic even though it has been successful in the experimental study [15]. In this regard, a successful case of intravenous hemofiltration treatment in ASE thrombectomy was first reported by Mutirangura et al [17] However, due to the limitation of intraoperative condition, postoperative continuous renal replacement therapy, i.e. continuous blood purification (CBP), has become a popular approach to correct life-threatening homeostatic disorders including metabolic acidosis, hyperkalemia, azotemia, and fluid overload. Therefore, initiation of renal replacement therapy at early stage may not only reduce the damage of kidney and other organs by acidosis, azotemia, fluid overload, and systemic inflammation, but also increase the survival rate and promote renal function recovery [18].

In the current study, surgical operation for ASE was started by indwelling 6F sheath at femoral venous on both sides. Through the sheath, venous blood (approximately 600 mL in total) was continuously drained into a collection bag during the process of arterial thrombus removal. After being processed by autologous blood recovery unit, intact erythrocytes were then transfused back to the patient. This procedure eliminated large amounts of toxic metabolites in the venous blood and thus, it minimized ischemia-reperfusion injury in the limbs. It was found that the oxygen carrying capacity of red blood cells in the autologous transfused blood was much higher, while amount of acidic substances was less, than that of the transfused blood from other donors. Hemostatic disorder, however, might occur if a large amount (3000 mL) of autologous blood were transfused [19, 20].

Considering the critical condition and unstable circulation of ASE patients, our experience indicated that it is critical to start early in draining venous blood of the lower limbs in order to reduce circulation of the metabolic toxins. In this regard, cardiac arrest and sudden death occurred in the two deceased patients of the current study before CBP was initiated due to the rapid progress of the disease. One of them died from sudden myocardial infarction and cardiac arrest 4 h after the operation, and the other one had cardiac arrest 20 min after the operation and died 6 h after the surgical operation. In contrast, the patient #2, who was 28 years old but suffered from ASE for 96 h, survived and was discharged from hospital after being successfully given CBP treatment. In these ASE patients with rapid deterioration of homeostasis during the surgical operation, we recognized that application of the arterial or venous sheath during surgical procedure may have the following advantages. 1). Operation time could be shortened by suturing artery puncture point after the patient’s condition is stable. 2). Acute reperfusion injury caused by sudden and rapid open blood flow could be avoided by the 8F arterial sheath through controlling arterial blood flow. 3). With the sheath in place, the patient is ready to receive CBP treatment immediately after he/she is transferred to the intensive care unit, which maximizes the opportunity of survival for the patient.

## Conclusion

In conclusion, ASE is a serious disease in the field of vascular surgery that threats patients’ life with high postoperative comorbidity and mortality. In clinical practice, open surgical thrombectomy is still the main method for ASE surgical treatment. However, PMT treatment for the ASE through Rotarex catheter is a minimally invasive whole lumen procedure with a high success rate technically. Advantages of PMT treatment includ labor saving, simple and speedy operation, and rapid restoration of blood flow. It is a fast, safe and feasible method. However, advanced technology does not always result in success in saving patients’ lives, and reperfusion injury is still a challenging issue in the treatment of severe limb ischemia. Continuous renal replacement therapy at early stage of postoperative care has been gradually recognized and applied in clinic, which will be of great significance in reducing relevant mortality and amputation rate.

## Abbreviations

ASE: Aorta saddle embolism
PMT: Percutaneous mechanical thrombectomy
TnT: Cardiac troponin T
CDT: Catheter-directed thrombolysis
MNMS: Myonephropathic metabolic syndrome
CK: Creatine phosphokinase
CBP: Continuous blood purification
